# Effect of immediate initiation of invasive ventilation on mortality in acute hypoxemic respiratory failure: a target trial emulation

**DOI:** 10.1186/s13054-024-04926-y

**Published:** 2024-05-10

**Authors:** Ricard Mellado-Artigas, Xavier Borrat, Bruno L. Ferreyro, Christopher Yarnell, Sicheng Hao, Kerollos N. Wanis, Enric Barbeta, Antoni Torres, Carlos Ferrando, Laurent Brochard

**Affiliations:** 1 Surgical Intensive Care Unit, Hospital Clínic de Barcelona, Barcelona, Spain; 2 CIBER de Enfermedades Respiratorias (CIBERES), Instituto de Salud Carlos III, Madrid, Spain; 3 Institut d’Investigacions Biomèdiques August Pi I Sunyer (IDIBAPS), Barcelona, Spain; 4 Keenan Research Centre for Biomedical Science, Li Ka Shing Knowledge Institute, St Michael’s Hospital, Unity Health Toronto, Toronto, ON Canada; 5 Interdepartmental Division of Critical Care Medicine, University of Toronto, Toronto, Canada; 6 Division of Respirology, Department of Medicine, University Health Network and Sinai Health System, Toronto, Canada; 7 Department of Critical Care Medicine, Scarborough Health Network, Toronto, ON Canada; 8 MIT IMES: Massachussetts Institute of Technology Institute for Medical Engineering and Science, Cambridge, USA; 9 Department of Breast Surgical Oncology, The University of Texas MD Anderson Cancer Center, Houston, TX USA; 10 Respiratory Intensive Care Unit, Pneumology, Respiratory Institute, Hospital Clinic of Barcelona, Barcelona, Spain

**Keywords:** Critical care, Respiratory insufficiency, Artificial respiration

## Abstract

**Purpose:**

Invasive ventilation is a fundamental treatment in intensive care but its precise timing is difficult to determine. This study aims at assessing the effect of initiating invasive ventilation versus waiting, in patients with hypoxemic respiratory failure without immediate reason for intubation on one-year mortality.

**Methods:**

Emulation of a target trial to estimate the benefit of immediately initiating invasive ventilation in hypoxemic respiratory failure, versus waiting, among patients within the first 48-h of hypoxemia. The eligible population included non-intubated patients with SpO_2_/FiO_2_ ≤ 200 and SpO_2_ ≤ 97%. The target trial was emulated using a single-center database (MIMIC-IV) which contains granular information about clinical status. The hourly probability to receive mechanical ventilation was continuously estimated. The hazard ratios for the primary outcome, one-year mortality, and the secondary outcome, 30-day mortality, were estimated using weighted Cox models with stabilized inverse probability weights used to adjust for measured confounding.

**Results:**

2996 Patients fulfilled the inclusion criteria of whom 792 were intubated within 48 h. Among the non-invasive support devices, the use of oxygen through facemask was the most common (75%). Compared to patients with the same probability of intubation but who were not intubated, intubation decreased the hazard of dying for the first year after ICU admission HR 0.81 (95% CI 0.68–0.96, *p* = 0.018). Intubation was associated with a 30-day mortality HR of 0.80 (95% CI 0.64–0.99, *p* = 0.046).

**Conclusion:**

The initiation of mechanical ventilation in patients with acute hypoxemic respiratory failure reduced the hazard of dying in this emulation of a target trial.

**Supplementary Information:**

The online version contains supplementary material available at 10.1186/s13054-024-04926-y.

## Background

Invasive ventilation represents a life-saving procedure and has been a key component of intensive care medicine for decades. Despite its benefits, its use is associated with peri-intubation complications, prolonged stay, acquired weakness, delirium and secondary infections [1–4]. For these reasons, there has been a growing interest to study the effectiveness of non-invasive strategies [5, 6].

The failure of non-invasive support carries a poor prognosis in acute respiratory failure [7–11], suggesting that one potential mechanism of harm could be related to spontaneous ventilation being injurious in situations where lungs have been primed for injury and strong breathing efforts take place [12]. Nonetheless, the decision to intubate is not always straightforward given the potential opposing complications associated with invasive ventilation and the risks of delaying intubation. This seems to be case especially in patients with severe hypoxemia but without impending signs of exhaustion. Perhaps for this reason, literature has shown a high degree of variability among clinicians regarding the institution of mechanical ventilation [13, 14]. Moreover, to this date there is no high-quality data to inform the best timing to initiate invasive ventilation since trials comparing a strategy of withholding intubation and another approach of carrying out expeditious intubation have never been carried out given feasibility reasons. In settings like this one, using observational data to emulate a potential trial can provide useful information about the potential benefit of a treatment at study [15].

Several observational studies have shown that delaying intubation can be associated with an increased mortality [8, 10, 11, 16–18]. However, in their comparisons these studies have largely ignored the population of patients who never ended up receiving intubation, thus making it difficult to draw firm conclusions about the optimal timing of instituting mechanical ventilation to large samples of patients with respiratory failure [19, 20]. In studies where data were used to emulate a potential trial comparing initiation of mechanical ventilation to non-invasive treatment, the results were not able to confirm that the former was superior to the latter, suggesting that invasive ventilation should be best regarded as a rescue therapy in patients failing non-invasive support [21–24]. This is an important discussion because this “wait and see” approach could spare many patients from an aggressive approach while maintaining its life-saving benefits at a population level.

However, newer real-world datasets have emerged in the last few years where information regarding patients’ status is available hourly after ICU admission [25]. With this in hand, researchers can more effectively adjust for confounding by indication and attempt to estimate the potential benefit of intubation in acute hypoxemic respiratory failure. In this study, we aimed at evaluating, every hour, within the first 48 h, the estimated treatment effect of starting invasive ventilation versus a strategy of trying to buy time on one-year mortality in patients with de novo hypoxemic respiratory failure using data from the Mart for Intensive Care-IV (MIMIC-IV) database, after adjusting for measured confounding [25, 26].

## Methods

This study represents an analysis of a real-world dataset, the Medical Information Mart for Intensive Care-IV (MIMIC-IV), that was created by the Massachussets Institute of Technology (MIT) and provides critical care data for over 60,000 patients admitted to intensive care units at the Beth Israel Deaconess Medical Center (BIDMC) between 2008 and 2019 [25, 26]. This dataset provides granular information on demographics as well as many physiological variables, treatment received and mortality up to 1-year post-discharge. This study was conducted following the standards as defined by the Declaration of Helsinki. Since MIMIC-IV only includes anonymized information, patients’ consent to participate was waived at the local institution. The Research Ethics Board at Hospital Clinic in Barcelona did not require to undergo further protocol approval.

### Eligibility criteria for the emulated trial

Patients were considered eligible if they had been admitted to the Medical, Medical/Surgical or Coronary ICUs and presented with acute hypoxemic respiratory failure, as defined by a ratio of oxygen saturation (SpO_2_) to inspired oxygen fraction (FiO_2_) ≤ 200 and a SpO_2_ ≤ 97% within 48 h of ICU admission and were not yet intubated. Patients could be receiving oxygen through facemask, high flow nasal cannula or non-invasive ventilation. We also wanted to exclude patients with immediate and major reason for endotracheal intubation. Therefore, exclusion criteria were a respiratory rate > 39 breaths per minute, a Glasgow Coma Scale ≤ 12 or a SpO_2_/FiO_2_ < 88 and the absence of a “Full Code”. These criteria were created to provide realistic limits to the inclusion of patients, since equipoise regarding withholding intubation would likely not hold in the latter subset.

### Target trial emulation

To estimate the effect of immediately initiating invasive ventilation on survival in patients with hypoxemic respiratory failure without prior history of intubation during the ICU admission, we emulated a target trial comparing intubation within one hour versus delaying intubation. Patients were eligible for the target trial in the first hour that they met eligibility criteria and for every subsequent hour in which they also met eligibility criteria, up to 48 h (Additional file 1: Table S1). This arbitrary time point was chosen because most intubations occur during this period and to provide greater homogeneity between patients.

**Fig. 1:**
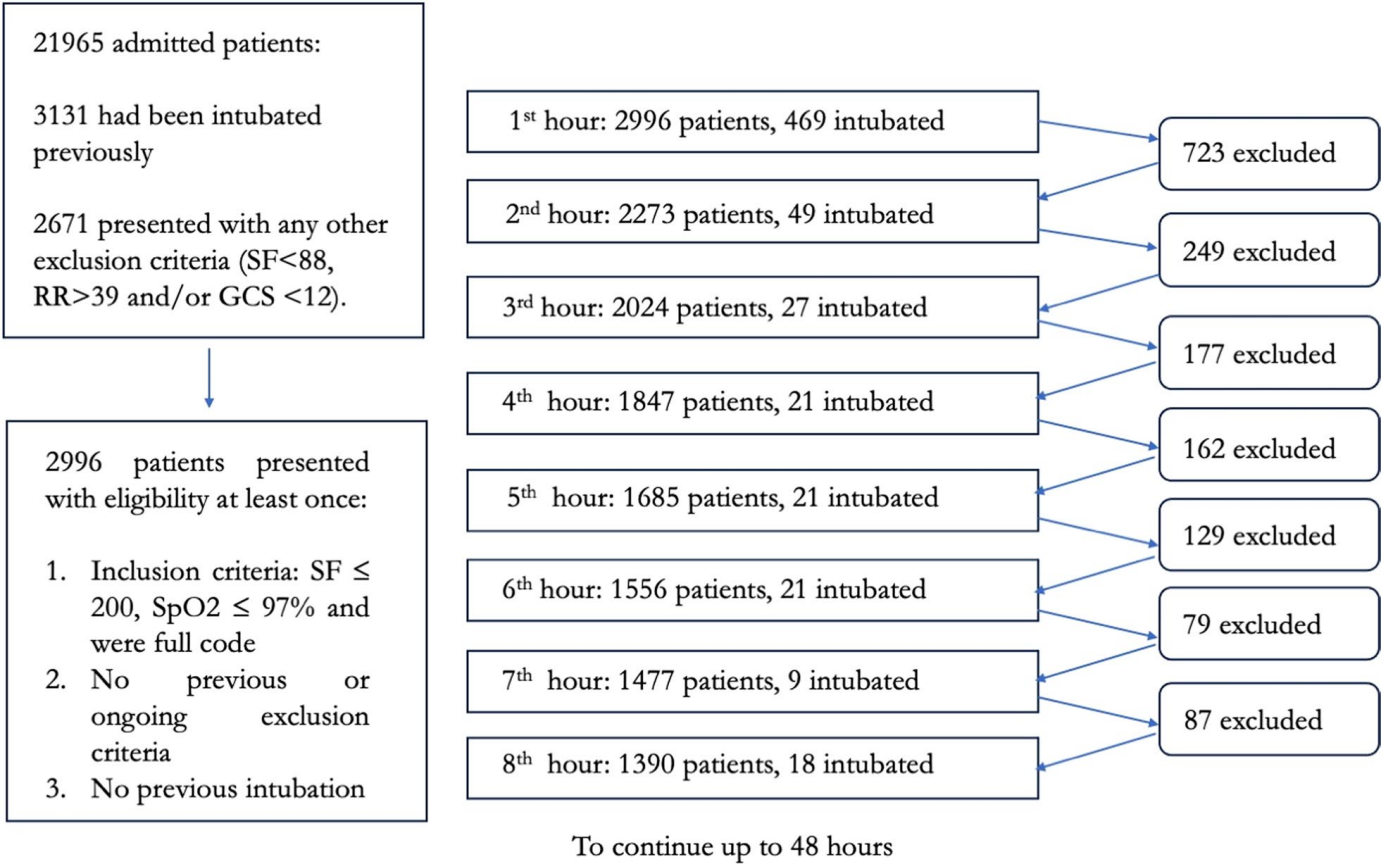
Study flowchart. Patients could be included if they had been admitted in any of the following ICUs: Medical, Medical/Surgical or Coronary ICU, had not been intubated previously and did not present any exclusion criteria. Afterwards, if they presented with all the inclusion criteria, it was considered that they had met eligibility and they were included in target trial number 1. Each patient could later contribute to future observations in the following 48 h, provided he/she did not receive intubation in the current target trial and that he/she continued to present eligibility in the following hours. For example, 723 patients were excluded from target trial number 2 with 469 patients having received intubation and 254 patients not presenting with further eligibility (either because of any new exclusion criteria, not further inclusion criteria or both). A total of 38,272 patient-observations were included of which 747 corresponded to observations where intubation took place. SF: SpO_2_/FiO_2_, RR: respiratory rate, GCS: Glasgow Coma Scale

To emulate the target trial, we identified all subjects that fulfilled the inclusion criteria (and this was considered the time that eligibility had been first met, or hour 1). This procedure was repeated throughout hours 2–48 for all remaining eligible patients who had not received invasive ventilation previously. At each hour to still be considered eligible, patients had to remain non-intubated at the beginning of the interval and had to continue to fulfill the inclusion criteria as well as not to fulfil any of the exclusion criteria. Thus a patient who remained eligible and non-intubated could contribute up to 48 observations to the target trial emulation [27] (Fig. 1). This methodology was followed to aim at reproducing what often happens in the clinical setting where clinicians continuously reassess their patients regarding the decision for intubation.

### Outcomes

The main outcome evaluated on this study was one-year mortality while, 30-day mortality, ICU and hospital length of stay were defined as secondary outcomes.

### Missing data

When missing data was present at any given hour after first eligibility, last observation carried forward was used for physiological data; under the assumption that physiological data would not deviate significantly from a previous value unless there existed a new entry in patients’ charts (see Additional file 1).

### Statistical analysis

At each evaluated time point throughout hour 1 to hour 48, patients’ probability to receive mechanical ventilation was estimated. To calculate this, a logistic regression with the receipt of mechanical ventilation as the dependent variable and variables supposed to play a role in the decision for intubation were used as independent variables. The variables included the time since fulfilling the inclusion criteria, age, comorbidities as measured by the Elixhauser comorbidity index, FiO_2_, SpO_2_/FiO_2_, respiratory rate, Glasgow Coma Scale, the use of any vasopressors and the admitting unit. After this propensity score had been estimated, stabilized inverse probability weights (IPW) were computed to adjust for confounding (see Additional file 1) [28, 29]. This approach resulted in a population that was weighted at each hour by their probability of receiving intubation [19, 30].

On this population, one-year mortality was later assessed in a time-to-event fashion with the use of a weighted Cox model. This model also accounted for systolic, median and diastolic blood pressure, temperature, creatinine and bilirubin levels as well as platelet count because these values may influence mortality independently from the decision to intubate patients. Hazard ratios (HR) are reported as an average of treatment effect over the study time and survival curves were constructed using a stratified Cox model [31] (see Additional file 1). 95% confidence intervals were calculated by estimating robust standard errors to account for the multiplicity of same-subject observations [27]. Unadjusted and adjusted mortalities were calculated using survival probabilities estimated with a non-parametrically bootstrapped Cox model with 1000 repetitions. ICU and hospital length of stay were assessed using weighted medians (and interquartile ranges) after bootstrapping and differences between groups with their 95% confidence intervals are presented. Reported p-values are two-sided and the level of significance was set at 0.05.

### Sensitivity analysis

Several additional analyses were conducted in restricted populations or using different statistical methods for confounding adjustment. First, the inclusion criteria were tightened to include a population of patients that besides hypoxemia also presented with a ROX ≤ 4.88 at eligibility. This cut-off was had previously shown to predict intubation in patients with acute hypoxemic respiratory failure under high-flow oxygen therapy [32]. Second, the effect of time since eligibility was further evaluated considering nested target trials within 5 groups: first hour after first eligibility, 2nd to 6th hour, 7th to 12th, 13th to 24th and 25th to 48th hour. Third, we repeated the main analysis using two doubly robust approaches, one with augmented inverse probability weighting (AIPW) and a second one using targeted maximum likelihood (TMLE). Fourth, we carried out overlap IPW weighting to limit the analysis to subjects with a realistic probability of receiving either treatment under investigation. Fifth, we repeated the analysis by restricting to the Medical ICU only. Sixth, we conducted a complete-case analysis. This was done to check the robustness of our study findings (see Additional file 1).

### Data handling

To construct the dataset for this study Google BigQuery was connected to MIMIC-IV and the R software (R Foundation for Statistical Computing, Vienna, Austria) was used for statistical analysis. All the code is available at https://github.com/rmartigas/causal-inference-invasive-ventilation-MIMIC-IV.

## Results

A total of 2996 patients fulfilled eligibility criteria at a median of 4 (1–12) hours after ICU admission, of whom 792 (26%) received intubation within 48 h of meeting eligibility at a median of 0 (IQR 0–4) hours after first eligibility [Figs. 1, Additional file 1: Fig. S1 and Table S2].

This initial population contributed to a total of 38,353 patient-observations over 48 nested trials and individual subjects contributed to a median number of 6 (IQR 2–18) target trials each (Fig. 1 and Additional file 1: Tables S2 and S3). At first eligibility, subjects who received invasive ventilation were younger than those who did not [63 (16) vs 65 (16) years, *p* < 0.001] and displayed more comorbidities as measured by the Elixhauser Comorbidity Index [14 (9) vs 12 (9) points, *p* < 0.001]. Also, they were more hypoxemic [SpO_2_/FiO_2_ 142 (38) vs 157 (30), *p* < 0.001], received a higher oxygen concentration [72 (21) vs 62 (15) %, *p* < 0.001] and displayed a higher respiratory rate [24 (7) vs 24 (6) breaths per minute, *p* = 0.04]. Use of vasopressor was uncommon between groups [median 0 (IQR 0–0) mcg/kg/min in both groups] [Table 1]. Among the non-invasive support devices, the use of oxygen through facemask was the most common (75%) [Additional file 1: Figure S2]. Unadjusted mortality occurred in 341 (43.8%) patients who received intubation within 48h and 848 patients (38.2%) who did not (*p* = 0.008).

**Table 1:** Characteristics of patients who received and did not receive intubation within 48 h considering first time of eligibility (only information at hour 1 is selected)

		No intubation within 48 h (2217 patients)	Intubation within 48 h (779 patients)	p-value
Age	Mean (SD)	65.9 (16.2)	63.1 (15.8)	< 0.001
Elixhauser comorbidity index	Mean (SD)	12.3 (9.2)	13.8 (9.0)	< 0.001
Non-invasive ventilation at eligibility	No	2023 (91.2)	733 (94.1)	0.015
	Yes	194 (8.8)	46 (5.9)	
High Flow cannula at eligibility	No	2075 (93.6)	756 (97.0)	< 0.001
	Yes	142 (6.4)	23 (3.0)	
FiO_2_	Mean (SD)	62.2 (15.2)	71.5 (20.8)	< 0.001
SpO_2_/FiO_2_	Mean (SD)	157.3 (30.1)	141.6 (38.0)	< 0.001
Respiratory rate (rpm)	Mean (SD)	23.8 (6.2)	24.3 (6.5)	0.038
ROX index	Mean (SD)	7.2 (2.7)	6.3 (2.6)	< 0.001
Temperature	Mean (SD)	36.8 (0.7)	36.8 (0.8)	0.996
Heart rate	Mean (SD)	95.4 (20.3)	98.5 (21.7)	< 0.001
SBP (mmHg)	Mean (SD)	123.0 (22.7)	121.6 (24.7)	0.174
DBP (mmHg)	Mean (SD)	68.3 (17.5)	68.3 (19.8)	0.995
MBP (mmHg)	Mean (SD)	81.9 (17.8)	81.1 (19.3)	0.291
Vasopressor (mcg/kg/min)	Median (IQR)	0 (0–0)	0 (0–0)	< 0.001
GCS	12	56 (2.5)	14 (1.8)	0.387
	13	104 (4.7)	37 (4.7)	
	14	306 (13.8)	123 (15.8)	
	15	1751 (79.0)	605 (77.7)	
Bilirubin (mg/dL)	Mean (SD)	1.9 (4.1)	2.7 (5.8)	< 0.001
Creatinine (mg/dL)	Mean (SD)	1.6 (1.6)	1.9 (1.9)	< 0.001
Platelet count	Mean (SD)	219.2 (120.6)	211.7 (136.5)	0.148
Admitting unit	Coronary ICU	496 (22.4)	141 (18.1)	0.001
	Medical ICU	894 (40.3)	374 (48.0)	
	Medical/Surgical ICU	827 (37.3)	264 (33.9)	
*Outcomes*				
30-day mortality	Yes	547 (24%)	232 (29.8%)	< 0.001
One-year mortality	Yes	848 (38.2%)	341 (43.8%)	0.008

Continuous variables are presented as means (SD) and categorical variables are presented as counts and percentages. The Elixhauser Comorbidity Index is a method of categorizing comorbidities of patients based on the International Classification of Diseases (ICD). SpO_2_: oxygen saturation measured by pulsioximetry, FiO_2_: inspired oxygen fraction, ROX: ratio of SpO_2_/FiO_2_ by respiratory rate, SBP: systolic blood pressure, DBP: diastolic blood pressure, MBP: mean blood pressure, GCS: Glasgow Coma Scale

**Fig. 2:**
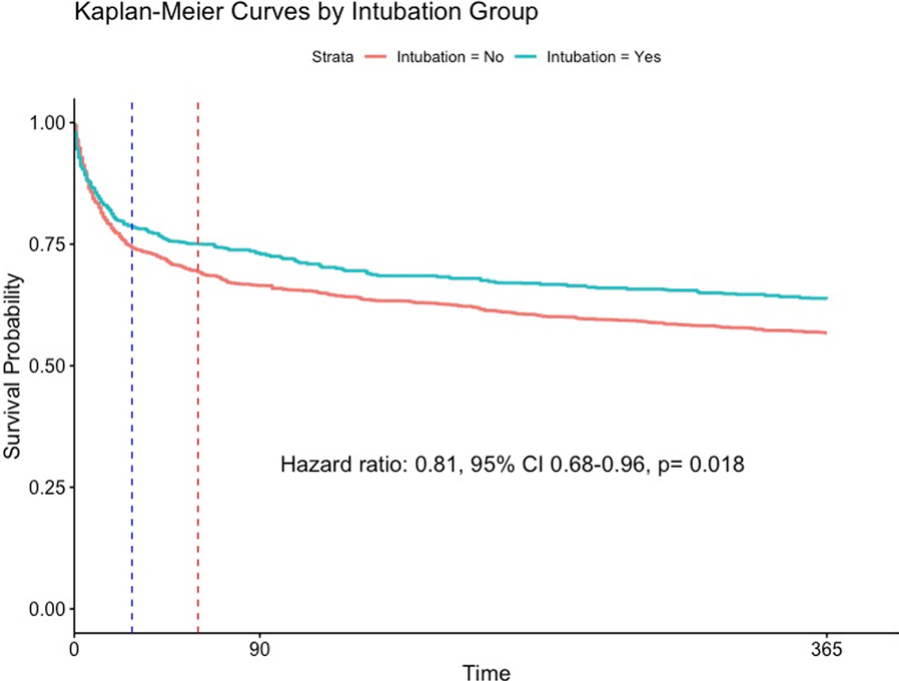
Survival curves estimated from the weighted Cox model. After IPW estimation, the population at study comprised of 38,272 patient-observations of whom 747 and 37,525 received and did not receive invasive ventilation. Kaplan–Meier curves for these weighted population showed that invasive ventilation was associated with a decreased hazard of dying over the following year. Dashed lines represent 28 and 60 days respectively after first-met eligibility

In the weighted population, intubation led to a decreased one-year mortality hazard ratio [HR 0.81 (95% CI 0.68–0.96, *p* = 0.018)] [Fig. 2 and Additional file 1: Figures S3 and S4]. By the end of this follow-up, mortality rate was 36% in intubated subjects and 43% in non-intubated subjects [absolute risk reduction 7%, (95% CI 3–11%)]. At one-month, unadjusted mortality had occurred in 232 (29.7%) and 547 (24%) for intubated and non-intubated subjects. After adjustment, the results showed that intubation was protective (HR 0.80, 95% CI 0.64–0.996, *p* = 0.046) with adjusted 30-day mortality rates being 20.4% and 25.4% [absolute risk reduction 5%, (95% CI 1.7–8.6%)].

**Fig. 3:**
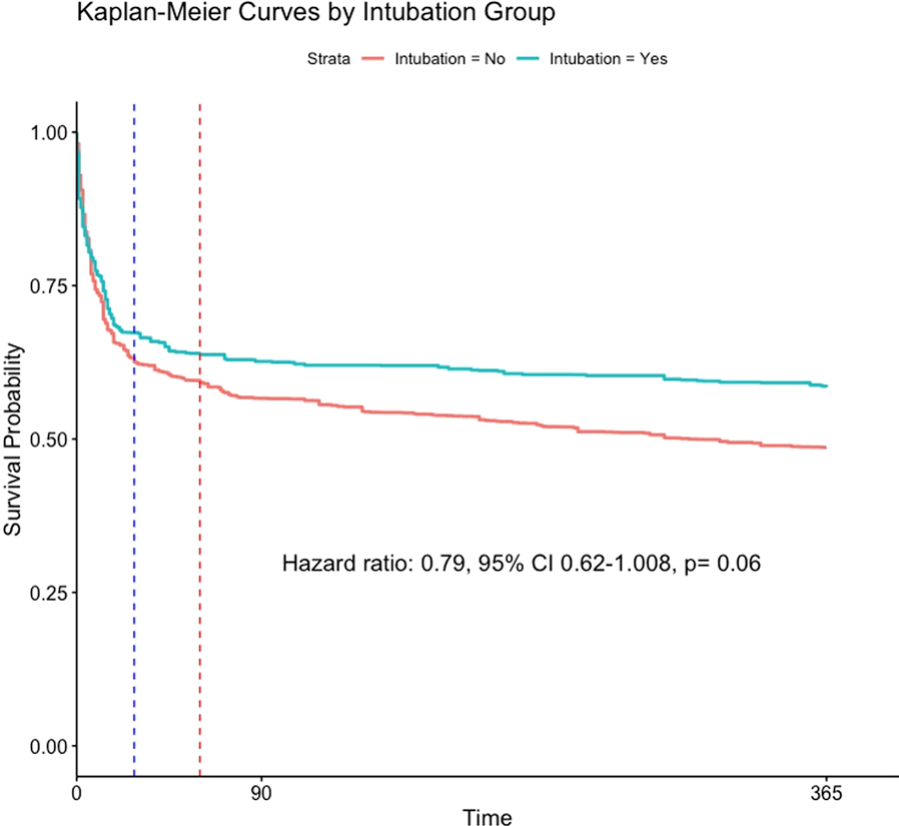
Kaplan–Meier curves for 7588 patient-observations with ROX ≤ 4.88 at eligibility of whom 348 were intubated. Dashed lines represent 28 and 60 days respectively

In a population of patients with a ROX ≤ 4.88, 1293 patients fulfilled eligibility in whom intubation within 48 h occurred in 348 (27%) individuals. Following the same nested design, this population led to 7588 patient-observations. Invasive ventilation followed the same direction (HR 0.79, 95% CI 0.62–1.008, *p* = 0.06) [Fig. 3 and Additional file 1: Table S4]. Adjusted one-year mortality rate was 41.3% in intubated subjects and 51.2% in non-intubated individuals [absolute risk reduction 9.9%, (95% CI 4.4–15.7%)].

Considering separate target trial emulations at each hour from the first hour of eligibility to the 48th did not show that time played any role in the potential benefit of instituting intubation (Table 2).

**Table 2:** Hazard ratios (HR) for treatment effect considering 5 different time windows since first eligibility

	1st h	2nd to 6th h	7th to 12th h	13th to 24th h	25th to 48th h
One-year mortality HR	0.87 (0.71–1.05)	1.008 (0.71–1.44)	1.008 (0.59–1.72)	1.13 (0.73–1.77)	0.85 (0.43–1.71)
30-day mortality HR	0.93 (0.74–1.17)	0.78 (0.47–1.31)	1.002 (0.48–2.11)	0.96 (0.53–1.74)	1.10 (0.54–2.24)

Further sensitivity analyses with AIPW provided an estimate for one-year OR of 0.86 (95% CI 0.79–0.93). One-year OR estimated with TMLE was non-significant (0.89, 95% CI 0.66–1.03). Overlap weights also led to non-significant results in the main cohort and in patients with ROX ≤ 4.88 only: for one-year mortality, HR was 0.93 (95% CI 0.82–1.05, *p* = 0.24) and for 30-day mortality, HR was 0.93 (95% CI 0.79–1.09, *p* = 0.35) [Additional file 1: Tables S7 and S8]. The estimated results by admitting unit can be seen in Additional file 1: Tables S5 and S6. The results of the complete case analysis can be seen in Additional file 1: Table S9.

The effect of intubation on ICU and hospital length of stay can be read in the Additional file 1: Table S10.

## Discussion

This study represents an analysis from a real-world dataset to emulate a target trial to assess the benefit of expeditious intubation in acute respiratory failure. To evaluate the outcomes, we adjusted for measured confounding using demographic and hourly physiological information as well as an index that captures the number and severity of comorbidities.

The study population was comprised of mostly middle-aged people and who were moderately comorbid at baseline. Their respiratory status showed moderate-to-profound hypoxemia without impending signs of decompensation since respiratory rate was, in general, well below 30 breaths per minute and Glasgow Coma Scale was preserved. Also, the use of vasopressor was uncommon in this cohort. The main result was that initiation of mechanical ventilation reduced the hazard of dying during a one-year follow-up by 20% and that this result was statistically significant. Several sensitivity analyses pointed to a reduction in the hazard ratio of around 15% during the first year. Moreover, when we assessed a population that beyond hypoxemia showed high respiratory rate as measured by a ROX ≤ 4.88, the results were consistent with the main estimate. When we restricted our analysis by ICU type, the estimates for each ICU were compatible with the main results.

To evaluate the impact of the timing of intubation on outcome, we split the population into several groups based on the moment since eligibility and no differences were identified in the estimates across different time points. However, we must note than most than 50% of intubations took place in the first hour after patients fulfilled the inclusion criteria and this might have reduced the precision of our estimates after this time point.

Another finding of this study was that intubation increased ICU and hospital length of stay by only 2 and 4 days, a difference that remained significant when we considered patients who survived ICU and hospital admission only.

Previous research has suggested that delaying intubation can worsen patients’ outcomes [8, 10, 11, 16–18]. Nonetheless, the methodology followed in these previous publications is, in our opinion, problematic because it compared only those patients who received mechanical ventilation. Patients who ultimately did not become intubated were not analyzed, thus limiting the ability to draw causal conclusions on the usefulness of implementing an aggressive approach towards initiating mechanical ventilation in acute hypoxemic respiratory failure [19, 20]. In contrast, more recent investigations emulating target trials to assess the benefit of initiating mechanical ventilation in both COVID-19 and septic shock patients were published, suggesting that *early* intubation does not improve outcomes in patients with respiratory failure but without impending signs of decompensation (highly elevated respiratory rate or low Glasgow Coma Scale). Nonetheless, several differences should be noted when comparing those previous works to the current research. First, the in-hospital mortality in the target trial conducted in COVID-19 patients was markedly lower (16%) than the 30-day mortality described in the current research [21]. Second, in the present study, patients were rarely in septic shock while in the aforementioned septic shock sub-study median vasopressor dose was around 0.5 mcg/kg/min. Indeed, the initiation of mechanical ventilation had a large hemodynamic effect in the previous study perhaps negatively affecting the results [22]. Finally, those previous publications were not able to use hourly data and only selected the worst values observed on longer periods of time such as 8 or 24 h unlike the current investigation, which we believe strengthens the robustness of our findings.

Recently, Yarnell et al. have emulated several trials aiming to identify different oxygenation thresholds. Using data from the MIMIC-IV, the investigators suggested that using a threshold of SpO_2_/FiO_2_ 110 as compared to 98 or 88 could decrease mortality [23]. In our current investigation, we decided to exclude patients with SpO_2_/FiO_2_ < 88 since it was felt that these subjects would likely be excluded of a potential clinical trial given that most physicians would intubate them right away. Also, using data from MIMIC-IV, Wanis and colleagues have recently published their analysis suggesting that invasive ventilation would not decrease in-hospital mortality as opposed to the use of non-invasive support [24]. Nonetheless, that study selected a wider population of patients where unadjusted 30-day mortality was lower (20.5%) and respiratory failure was not as tightly defined as in our research where patients were only included if they presented with a SpO_2_/FiO_2_ between 89 and 200 (with a SpO_2_ ≤ 97%). While Wanis’ and our results align in ruling out a harmful effect caused by early/expeditious intubation, the current findings reinforce the idea that if immediate intubation must prove beneficial this seems to be more likely the case the sicker the studied populations are.

This study deserves several considerations. First, the MIMIC-IV dataset comprises a single-center real-world cohort and data is electronically recorded and stored in a server. To avoid volatility in physiological recordings data was averaged hourly for most signals such as SpO_2_ and respiratory rate which might have removed some extreme but valuable information. Second, as with other observational studies, the finding in this study, that invasive ventilation decreased one-year mortality in a broad population of patients, is prone to unmeasured confounding. Third, the nature of the dataset did not allow to explore the primary diagnosis with certainty, and we aimed at analyzing patients admitted at the Medical, Medical/Surgical and Coronary ICU, who were non-intubated and who presented with acute hypoxemia within 48 h of admission. Fourth, parameters drawn from arterial blood gases were not assessed since missing data was large. Likewise, data regarding chest radiology could not be used. Finally, the current MIMIC-IV version does not include data on COVID-19 where the so-called early intubation has been repeatedly questioned [21, 33, 34]. Fifth, several sensitivity analyses using doubly robust methods as well as overlap weighting did not show that the use of immediate intubation would decrease the hazard of dying; however, these methods did not point to a signal for harm either. Further research using newer datasets might be able to offer more accurate information including COVID-19 respiratory failure management.

## Conclusions

This study represents an effort to estimate the potential impact of immediately starting invasive ventilation in patients recently admitted to the ICU with acute hypoxemic respiratory failure using observational data. After excluding patients with a major and immediate reason for intubation, the current research suggests that intubation might not only be a valuable rescue therapy in hypoxemic respiratory failure but that its early use might decrease mortality; however, several sensitivity analyses did not show significant results, limiting the robustness of the main study findings. Nonetheless, as with prior target trial emulations, the estimates provided in this manuscript do not point towards increased harm with intubation.

## Supplementary Information

**Additional file 1.** Supplementary tables and figures.

## Data Availability

All the code used for this analysis is publicly available.
