# Supplementary material for: Effect of immediate initiation of invasive ventilation on mortality in acute hypoxemic respiratory failure: a target trial emulation
Source: Crit Care. 2024 May 10;28:157. doi: 10.1186/s13054-024-04926-y (PMC11088053; doi:10.1186/s13054-024-04926-y)
Supplement: Supplementary file 1 — Additional file 1. Supplementary tables and figures. [file 13054_2024_4926_MOESM1_ESM.docx]

Effect of immediate initiation of invasive ventilation on mortality in acute hypoxemic respiratory failure: a target trial emulation

R Mellado-Artigas^1,2,3,4^, X Borrat^1^, BL Ferreyro^5,6^, C Yarnell^5,6,7^, S Hao^8^, K Wanis^9^, E Barbeta^1,2,3^, A Torres^2,3,10^, C Ferrando^1,2,3^, L Brochard^4,5^

1. Surgical Intensive Care Unit, Hospital Clínic de Barcelona, Barcelona, Spain.

2. CIBER de Enfermedades Respiratorias (CIBERES), Instituto de Salud Carlos III, Madrid, Spain.

3. Institut d’Investigacions Biomèdiques August Pi I Sunyer (IDIBAPS), Barcelona, Spain.

4. Keenan Research Centre for Biomedical Science, Li Ka Shing Knowledge Institute, St Michael's Hospital, Unity Health Toronto, Toronto, ON, Canada.

5. Interdepartmental Division of Critical Care Medicine, University of Toronto, Toronto, Canada

6. Department of Medicine, Division of Respirology, University Health Network and Sinai Health System, Toronto, Canada

7. Department of Critical Care Medicine, Scarborough Health Network, Toronto, ON, Canada

8. MIT IMES: Massachusetts Institute of Technology Institute for Medical Engineering and Science

9. Department of Breast Surgical Oncology, The University of Texas MD Anderson Cancer Center, Houston, Texas, USA

10. Respiratory Intensive Care Unit, Pneumology, Respiratory Institute, Hospital Clinic of Barcelona, Barcelona, Spain.

[FURTHER INFORMATION ON METHODS 1](#_Toc163569778)

[*ELIGIBILITY CRITERIA & TRIAL EMULATION* 1](#_Toc163569779)

[*STUDY OUTCOMES:* 3](#_Toc163569780)

[*DATA HANDLING & STATISTICAL ANALYSIS:* 4](#_Toc163569781)

[Table S1: Description of the target trial 7](#_Toc163569782)

[RESULTS 9](#_Toc163569783)

[Table S2: Study flowchart for each of the 48 hours 9](#_Toc163569784)

[Table S3. 12](#_Toc163569785)

[Table S4: Characteristics of patients who received and did not receive intubation within 48 hours considering first time of eligibility (only information at hour 1 is selected) in the cohort of patients with ROX equal or below to 4.88. 13](#_Toc163569786)

[Table S5: Results, expressed in hazard ratio scale, when restricting the population to specific ICUs. 14](#_Toc163569787)

[Table S6: Results at one-year by specific ICUs using doubly robust logistic regression and overlap weighting. AIPW: Augmented inverse probability weighting, TMLE: Targeted maximum likelihood. 14](#_Toc163569788)

[Table S7: Results using AIPW, TMLE and overlap weighting in the population with ROX ≤4.88 at eligibility. AIPW: Augmented inverse probability weighting, TMLE: Targeted maximum likelihood. 14](#_Toc163569789)

[Table S8: Results at one-year using overlap weights for the first hour of observation and for the remaining hours (hours 2 to 48). This was done since overlap was significantly worse from hour 2 and onwards. 14](#_Toc163569790)

[Table S9: Complete-case analyses: 1) without any type of imputation including last observation carried forward and 2) allowing last observation carried forward for SOFA score items. 15](#_Toc163569791)

[Table S10: ICU and hospital length of stay in the whole cohort and in survivors only. 15](#_Toc163569792)

[Figure S1. Cumulative number of intubations over the study period. 16](#_Toc163569793)

[Figure S2. Distribution of non-invasive support received at each hour after eligibility had been met. 17](#_Toc163569794)

[Figure S3: Propensity score distribution by groups of treatment at hour 1. 18](#_Toc163569795)

[Figure S4: Propensity score distribution by groups of treatment from hour 2 and onwards. 19](#_Toc163569796)

[REFERENCES 20](#_Toc163569797)

# **FURTHER INFORMATION ON METHODS**

This study represents a retrospective analysis of a real-world dataset, the Mart for Intensive Care-IV (MIMIC-IV) database, that was created by the Massachussets Institute of Technology (MIT) and provides critical care data for over 60,000 patients admitted to intensive care units at the Beth Israel Deaconess Medical Center (BIDMC)^1^. MIMIC-IV spans from 2008 to 2019 and includes information from the Emergency department as well. This dataset provides granular information on demographics, diagnosis and previous comorbidities as well as timestamped data on many physiological variables, treatment received. It also includes mortality up to one-year post-discharge which is linked to provincial registries.

## *ELIGIBILITY CRITERIA & TRIAL EMULATION*

The current study aims at evaluating the potential benefit of initiating mechanical ventilation in acute hypoxemic respiratory failure, using observational data. To emulate such a potential trial, we aimed to include patients how were non-intubated at ICU arrival and who were hypoxemic as defined by a ratio of oxygen saturation (SpO_2_) to inspired oxygen fraction (FiO_2_) equal or less than 200, altogether by a SpO_2_ of 97% or less. Also, we mandated that patients be full code since otherwise intubation could not take place in that particular patient. Finally, in order to ensure that the patients enrolled in this emulated trial reflected subjects with a primary diagnosis of respiratory failure, these findings had to occur within 48 hours of ICU admission. In summary, this would equate to the inclusion criteria in the potential clinical trial to be emulated (Table S1).

Regarding exclusion criteria, we acknowledged that some patients would likely be excluded from a potential randomized trial because intubation would have been deemed essential and would have eventually been conducted right away. Then, we decided to exclude patients who were extremely hypoxemic, and we defined this as a SpO_2_/FiO_2_ of less than 88, very tachypneic (respiratory rate above 39 breaths per minute) or drowsy or comatose (Glasgow Coma Scale below 12 points).

Eligible patients could be receiving oxygen trough facemask, high flow nasal cannula or non-invasive ventilation at the time of eligibility. Although facemask has shown to be inferior to the other two techniques in preventing intubation, it has not shown to worsen patients’ outcomes and thus it was selected in the main analysis.

Once patients fulfilled all the inclusion criteria and were not/had never fulfilled any exclusion criteria, we considered the subjects eligible for the first time (and this was labelled as hour 1 in the study). Up to that moment, patients were followed for 48 hours to evaluate if the intervention (intubation) had taken place or not. Again, this was done to emulate a potential clinical trial where the intervention would likely last for 48-72 hours and after that clinicians would be ready to switch back to the standard of care.

In order to emulate clinical practice as much as possible, we reckoned that the decision to intubate is an ongoing process that is based on changing physiological data. For this reason and to maximize the information available in the dataset we decided to create a nested design where each hour during the first 48 hours after first eligibility was considered a separate study. Nonetheless, in order to qualify for subsequent trials (from hour 2 to hour 48) patients had to remain non-intubated and still present with all the inclusion criteria and do not present with any exclusion criteria. If a particular patient did not present with all the inclusion criteria in certain time point, he/she would not be eligible at that hour but could be later included for an upcoming time point. However, once the patient had ever presented with any exclusion criteria (or had been intubated) the subject would no longer be available for any upcoming trials. In summary, this study ended comprising of 48 nested hourly trials where subjects could eventually participate.

In sensitivity analysis, the investigators changed the inclusion criteria to select patients with a ROX index ≤4.88 since ROX has been prospectively validated to predict intubation in respiratory failure when using high flow nasal cannula but up to now, no data is available to inform whether using ROX to decide on intubation need will improve patients’ outcomes.

## *STUDY OUTCOMES:*

The main outcome of this study was the hazard risk of mortality during a one-year period after study enrolment. Since MIMIC-IV offers information up to one year after hospital discharge, information occurring beyond 365 days after hospital admission was censored at one-year after ICU admission. The same was carried out for 30-day mortality.

ICU and hospital length of stay was evaluated by means of weighted medians and quantiles with the use of the *Laeken* package in R. The weights here represent the same stabilized weights derived from the propensity score at each hour. Given the multiplicity of same-subject observations, non-parametric bootstrapping was carried out.

## *DATA HANDLING & STATISTICAL ANALYSIS:*

The construction of this dataset querying MIMIC-IV was carried out in Google BigQuery using SQL and the result is a data frame organized in the long format with rows representing one-hour intervals since ICU admission. This way, each subject would have multiple observations (up to 96 hours since ICU admission) that contained physiological data and information regarding intubation status. Physiological data was aggregated in median values for the hourly interval and information regarding exposure represents Yes/No. An hourly interval where invasive ventilation is newly set to 1 (Yes) would mean that the treatment has been started but it is not possible to know the exact time point in the hourly interval where this occurred. This is potentially problematic given that physiological data might have been collected afterwards and thus represent post-exposure information. To overcome this problem, it was thus decided to move (lag) the physiological information one hour ahead. Although this approach allowed the investigators to confidently state that physiological data truly represents a pre-intubation status, this would have forced to eliminate the first hour of observation in the ICU for all the patients. To avoid so, we collected information on pre-ICU status from hour before to ICU admission. Finally, as a sensitivity check, the main analysis was repeated without lagging, which led to very similar results (HR: 0.82, 95% CI 0.68-0.97, p= 0.02).

For the current study, the investigators estimated hourly patients’ probability to receive mechanical ventilation was based on the aforementioned physiological data^2^. This probability was calculated for each subject and hourly until the end of the 48-hour period or until the patient had been started on invasive mechanical ventilation, had presented any exclusion criteria, were not presenting the inclusion criteria anymore, had died or had left the unit.

To estimate the propensity score, a logistic regression with the receipt of mechanical ventilation as the dependent variable and time since fulfilling the inclusion criteria, age, comorbidities as measured by the Elixhauser comorbidity index, FiO_2_, SpO_2_/FiO_2_, respiratory rate, Glasgow Coma Scale, the use of any vasopressors and the admitting unit were used. Where FiO_2_ was not available, it was estimated via the oxygen flow with the use of the formula: 0.21 + (oxygen flow in liters per minute)* 0.03^3^. To account for trends in data, the propensity score from hour 2 and onwards also incorporated the previous hour values of FiO_2_, SpO_2_/FiO_2_ and respiratory rate. For each hourly observation, each patient presented with a probability between 0 and 1 to receive intubation. Later on, these probabilities were used to estimate stabilized inverse probability weights (IPW). ^4,5^. Since this approach carries the risk of inducing weight inflation in outliers, weight truncation was performed at percentile 99 and above. This allowed to maintain precision in the estimates while reducing standard errors.

Last observation carried forward was applied to the dataset assuming that most missing data would have reflected the absence of any meaningful change as significant deviations are more likely to be charted. After this, the amount of missing data was minimal as shown below:

1. For systolic median and diastolic blood pressure missing data was present in 62 observations (out of 38553, 0.001%). For heart rate data was missing in 13 observations.
2. For creatinine and platelets, data was missing in 11 observations and for bilirubin it was missing in 16 observations.

In cases where missing was still available, imputation with Markov chains with the use of the *mice* package was carried out. To do so, each missing value was imputed 25 times and the median of the imputations was selected. A descriptive analysis was carried out to evaluate that the distribution of the imputed variables and their correlation with others had not drifted from the pre-imputation values.

Finally, we conducted two different complete-case analysis. In the first one, we did not conduct last observation carried forward (LOCF) nor imputation with Markov chains.

One-year mortality was assessed in a time-to-event fashion with the use of a weighted Cox model (including the stabilized IPW weights) and further adjusting for heart rate, systolic, diastolic and mean blood pressure, temperature and non-respiratory Sequential Organ Failure Assessment (SOFA) score items. Hazard ratios (HR) are reported as an average of treatment effect over the study time and survival curves were constructed using the estimates from the Cox model^6^. 95% confidence intervals were calculated by estimating robust standard errors to account for the multiplicity of same-subject observations. Also, we decided to perform two doubly robust estimators: 1) augmented inverse probability weighting and 2) targeted maximum likelihood. Since our dataset did not present loss to follow-up, we calculated those estimators with logistic regression given that computational requirements were significantly lower this way. For the exposure model we used the same variables as described in the estimation of IPW and we added heart rate, systolic, diastolic and mean blood pressure, temperature and non-respiratory Sequential Organ Failure Assessment (SOFA) score items to them in the outcome model. We ran these analyses with AIPW and TMLE packages, the first one providing confidence intervals with standard errors after cross-validation with 10 folds and the second one with non-parametric bootstrapping with 1000 repetitions. Since the output of the first result provided much smaller confidence intervals, we cross-checked this finding (the one with AIPW) running this function in 100 bootstrapped repetitions that confirmed the provided estimate. Finally, given that overlap seemed to be rare after the second hour, we decided to perform overlap weighting for all the sub-analyses of this study.

## Table S1: Description of the target trial

|  | **Potential trial** | **Emulated trial** |
| --- | --- | --- |
| Population | Inclusion criteria: Patients admitted to the Medical ICU, Medical/Surgical ICU or Coronary ICU and presenting with SpO_2_/FiO_2_ ≤200 and SpO_2_ ≤97% within 48 hours of ICU admission.  Exclusion criteria: SpO_2_/FiO_2_ <88, respiratory rate > 39 bpm or GCS <12. | Inclusion criteria: Patients admitted to the Medical ICU, Medical/Surgical ICU or Coronary ICU and presenting with SpO_2_/FiO_2_ ≤200 and SpO_2_ ≤97% within 48 hours of ICU admission.  Exclusion criteria: SpO_2_/FiO_2_ <88, respiratory rate > 39 bpm or GCS <12. |
| Intervention | Intubation | Intubation within one hour |
| Comparison | Non-intubation unless any exclusion criteria arise. | Non-intubation unless any exclusion criteria arise. |
| Study design | Randomization at the time of eligibility. Intubation to be conducted as soon as possible. Protocol violation if intubation did not occur within one hour of randomization to intubation group. | Identification of patients at first eligibility (target trial 1). Afterwards, at each hour for 48 hours will still be if they have continued to not be intubated and still present with all the inclusion criteria and no exclusion criteria. This design can be considered to comprise 48 nested trials. |
| Outcome | One-year mortality risk (hazard ratio)  30-day mortality (hazard ratio)  ICU and hospital length of stay | One-year mortality risk (hazard ratio)  30-day mortality (hazard ratio)  ICU and hospital length of stay |
| Causal contrast | Intention to treat | Intention to treat |
| Statistical analysis | Cox model for one-year and 30-day mortality (hazard ratio).  Length of stay assessed by median difference. P-value assessed by Wilcoxon-Mann-Whitney. | In each trial, estimation of the probability of receiving intubation and construction of stabilized inverse probability of treatment weights.  Weighted Cox model for main outcome. Survival curves derived from stratified (by treatment groups) Cox models.  Median difference (and quantiles) via bootstrapping for length of stay. |

# **RESULTS**

Table S2: Study flowchart for each of the 48 hours (the 8 first hours are also presented in Figure 1 in the main text). At each hour, the number of patients intubated and non-intubated is presented. We used hour 1 as first hour after eligibility.

| **Hours since eligibility** | **Intubation** | **Case count** |
| --- | --- | --- |
| 1 | 0 | 2527 |
| 1 | 1 | 469 |
| 2 | 0 | 2224 |
| 2 | 1 | 49 |
| 3 | 0 | 1997 |
| 3 | 1 | 27 |
| 4 | 0 | 1826 |
| 4 | 1 | 21 |
| 5 | 0 | 1685 |
| 5 | 1 | 21 |
| 6 | 0 | 1566 |
| 6 | 1 | 21 |
| 7 | 0 | 1468 |
| 7 | 1 | 9 |
| 8 | 0 | 1372 |
| 8 | 1 | 18 |
| 9 | 0 | 1282 |
| 9 | 1 | 13 |
| 10 | 0 | 1213 |
| 10 | 1 | 10 |
| 11 | 0 | 1153 |
| 11 | 1 | 4 |
| 12 | 0 | 1087 |
| 12 | 1 | 8 |
| 13 | 0 | 1006 |
| 13 | 1 | 9 |
| 14 | 0 | 949 |
| 14 | 1 | 11 |
| 15 | 0 | 894 |
| 15 | 1 | 9 |
| 16 | 0 | 857 |
| 16 | 1 | 5 |
| 17 | 0 | 808 |
| 17 | 1 | 7 |
| 18 | 0 | 769 |
| 18 | 1 | 4 |
| 19 | 0 | 737 |
| 19 | 1 | 7 |
| 20 | 0 | 709 |
| 20 | 1 | 7 |
| 21 | 0 | 680 |
| 21 | 1 | 4 |
| 22 | 0 | 638 |
| 22 | 1 | 7 |
| 23 | 0 | 615 |
| 23 | 1 | 2 |
| 24 | 0 | 596 |
| 24 | 1 | 1 |
| 25 | 0 | 570 |
| 25 | 1 | 4 |
| 26 | 0 | 549 |
| 26 | 1 | 4 |
| 27 | 0 | 524 |
| 27 | 1 | 1 |
| 28 | 0 | 504 |
| 28 | 1 | 1 |
| 29 | 0 | 485 |
| 30 | 0 | 467 |
| 30 | 1 | 3 |
| 31 | 0 | 444 |
| 31 | 1 | 1 |
| 32 | 0 | 421 |
| 32 | 1 | 3 |
| 33 | 0 | 399 |
| 33 | 1 | 1 |
| 34 | 0 | 382 |
| 34 | 1 | 6 |
| 35 | 0 | 370 |
| 35 | 1 | 1 |
| 36 | 0 | 356 |
| 37 | 0 | 345 |
| 38 | 0 | 332 |
| 39 | 0 | 321 |
| 39 | 1 | 1 |
| 40 | 0 | 314 |
| 41 | 0 | 304 |
| 41 | 1 | 1 |
| 42 | 0 | 296 |
| 43 | 0 | 286 |
| 43 | 1 | 3 |
| 44 | 0 | 271 |
| 44 | 1 | 2 |
| 45 | 0 | 258 |
| 45 | 1 | 1 |
| 46 | 0 | 245 |
| 47 | 0 | 240 |
| 47 | 1 | 2 |
| 48 | 0 | 233 |
| 48 | 1 | 1 |

Table S3. Characteristics of the unadjusted population over the study period. Continuous variables are presented as means (SD) or medians (IQR) and categorical variables are presented as counts and percentages. The Elixhauser Comorbidity Index is a method of categorizing comorbidities of patients based on the International Classification of Diseases (ICD). SpO_2_: oxygen saturation measured by pulse oximetry, FiO_2_: inspired oxygen fraction, ROX: ratio of SpO_2_/FiO_2_ by respiratory rate, SBP: systolic blood pressure, DBP: diastolic blood pressure, MBP: mean blood pressure, GCS: Glasgow Coma Scale. P-values are presented after univariate after conducting tests for categorical or continuous variables.

|  |  | **No intubation (37561 patient-observations)** | **Intubation**  **(792 patient-observations)** | **p-value** |
| --- | --- | --- | --- | --- |
| Age | Mean (SD) | 65.7 (15.9) | 63.1 (15.8) | <0.001 |
| Elixhauser comorbidity index | Mean (SD) | 12.8 (9.0) | 13.8 (9.0) | 0.001 |
| Non-invasive ventilation at eligibility | No | 33844 (90.1) | 743 (95.4) | <0.001 |
|  | Yes | 3730 (9.9) | 36 (4.6) |  |
| High Flow cannula at eligibility | No | 31988 (85.1) | 762 (97.8) | <0.001 |
|  | Yes | 5586 (14.9) | 17 (2.2) |  |
| FiO_2_ | Mean (SD) | 64.2 (15.2) | 78.8 (22.4) | <0.001 |
| SpO_2_/ FiO_2_ | Mean (SD) | 152.3 (30.0) | 130.5 (41.3) | <0.001 |
| Respiratory rate (rpm) | Mean (SD) | 22.6 (5.9) | 24.1 (6.7) | <0.001 |
| ROX index | Mean (SD) | 7.3 (2.6) | 6.0 (2.9) | <0.001 |
| Heart rate | Mean (SD) | 90.9 (18.5) | 98.5 (22.3) | <0.001 |
| SBP (mmHg) | Mean (SD) | 119.5 (21.3) | 121.2 (25.1) | 0.029 |
| DBP (mmHg) | Mean (SD) | 64.9 (15.8) | 68.0 (20.1) | <0.001 |
| MBP (mmHg) | Mean (SD) | 78.3 (15.7) | 80.9 (19.7) | <0.001 |
| Vasopressor (mcg/kg/min) | Median (IQR) | 0 (0-0) | 0 (0-0) | <0.001 |
| GCS | 12 | 597 (1.6) | 12 (1.5) | 0.352 |
|  | 13 | 1552 (4.1) | 42 (5.4) |  |
|  | 14 | 5491 (14.6) | 117 (15.0) |  |
|  | 15 | 29934 (79.7) | 608 (78.0) |  |
| Bilirubin (mg/dL) | Mean (SD) | 1.8 (4.0) | 2.7 (5.9) | <0.001 |
| Creatinine (mg/dL) | Mean (SD) | 1.4 (1.4) | 1.9 (1.9) | <0.001 |
| Platelet count | Mean (SD) | 224.2 (123.7) | 210.4 (135.9) | 0.002 |
| Admitting unit | Coronary ICU | 7254 (19.3) | 141 (18.1) | <0.001 |
|  | Medical ICU | 15415 (41.0) | 374 (48.0) |  |
|  | Medical/Surgical ICU | 14905 (39.7) | 264 (33.9) |  |
| **Outcomes** |  |  |  |  |
| 30-day mortality | Yes | 10091 (26.9%) | 232 (29.8%) | 0.07 |
| One-year mortality | Yes | 16488 (43.9%) | 341 (43.8%) | 0.98 |

Table S4: Characteristics of patients who received and did not receive intubation within 48 hours considering first time of eligibility (only information at hour 1 is selected) in the cohort of patients with ROX equal or below to 4.88. Continuous variables are presented as means (SD) and categorical variables are presented as counts and percentages. The Elixhauser Comorbidity Index is a method of categorizing comorbidities of patients based on the International Classification of Diseases (ICD). SpO_2_: oxygen saturation measured by pulsioximetry, FiO_2_: inspired oxygen fraction, ROX: ratio of SpO_2_/FiO_2_ by respiratory rate, SBP: systolic blood pressure, DBP: diastolic blood pressure, MBP: mean blood pressure, GCS: Glasgow Coma Scale.

|  |  | **No intubation (943 patients)** | **Intubation**  **(346 patients)** | **p-value** |
| --- | --- | --- | --- | --- |
| Age | Mean (SD) | 65.3 (16.9) | 62.4 (15.8) | 0.005 |
| Elixhauser comorbidity index | Mean (SD) | 12.6 (9.0) | 14.1 (8.9) | 0.005 |
| Non-invasive ventilation at eligibility | No | 837 (11.2) | 316 (91.3) | <0.219 |
|  | Yes | 106 (88.8) | 30 (8.7) |  |
| High Flow cannula at eligibility | No | 803 (85.2) | 325 (93.9) | <0.001 |
|  | Yes | 140 (14.8) | 21 (6.1) |  |
| FiO_2_ | Median (IQR) | 66 (66 to 100) | 100 (70 to 100) | <0.001 |
| SpO_2_/ FiO_2_ | Mean (SD) | 125.5 (26.5) | 108.4 (22.7) | <0.001 |
| Respiratory rate (rpm) | Mean (SD) | 30.0 (5.2) | 29.0 (5.3) | 0.001 |
| ROX index | Mean (SD) | 4.2 (0.6) | 3.8 (0.7) | <0.001 |
| Heart rate | Mean (SD) | 99.8 (20.0) | 103.4 (21.4) | 0.005 |
| SBP (mmHg) | Mean (SD) | 124.1 (22.9) | 123.5 (24.9) | 0.677 |
| DBP (mmHg) | Mean (SD) | 69.9 (17.9) | 70.6 (21.1) | 0.605 |
| MBP (mmHg) | Mean (SD) | 83.5 (17.8) | 82.8 (20.4) | 0.543 |
| Vasopressor (mcg/kg/min) | Mean (SD) | 0.0 (0.0) | 0.0 (0.2) | 0.001 |
| GCS | 12 | 16 (1.7) | 5 (1.4) | 0.066 |
|  | 13 | 38 (4.0) | 20 (5.8) |  |
|  | 14 | 121 (12.8) | 61 (17.6) |  |
|  | 15 | 768 (81.4) | 260 (75.1) |  |
| Bilirubin (mg/dL) | Mean (SD) | 1.8 (4.1) | 2.6 (5.4) | 0.007 |
| Creatinine (mg/dL) | Mean (SD) | 1.4 (1.3) | 1.8 (1.8) | <0.001 |
| Platelet count | Mean (SD) | 227.0 (129.2) | 202.2 (136.2) | 0.003 |
| Admitting unit | Coronary ICU | 188 (19.9) | 55 (15.9) | 0.064 |
|  | Medical ICU | 393 (41.7) | 168 (48.6) |  |
|  | Medical/Surgical ICU | 362 (38.4) | 123 (35.5) |  |
| **Outcomes** |  |  |  |  |
| 30-day mortality | Yes | 292 (31%) | 129 (37.2%) | 0.04 |
| One-year mortality | Yes | 429 (45.5%) | 173 (50%) | 0.17 |

## Table S5: Results, expressed in hazard ratio scale, when restricting the population to specific ICUs.

|  | **30-day mortality** | **One-year mortality** |
| --- | --- | --- |
| **Medical ICU (1268 patients)** | 0.79 (0.57-1.08) | 0.81 (0.63-1.04) |
| **Medical/Surgical ICU (1091 patients)** | 0.76 (0.55-1.07) | 0.78 (0.59-1.03) |
| **Coronary ICU (637 patients)** | 0.87 (0.41-1.82) | 0.89 (0.54-1.46) |

## Table S6: Results at one-year by specific ICUs using doubly robust logistic regression and overlap weighting. AIPW: Augmented inverse probability weighting, TMLE: Targeted maximum likelihood.

|  | **AIPW** | **TMLE** | **Overlap weighting** |
| --- | --- | --- | --- |
| **Medical ICU (1268 patients)** | OR 0.81 (0.72-0.92) | OR 0.71 (0.51-0.98) | HR 0.95 (0.72-1.25) |
| **Medical/Surgical ICU (1091 patients)** | OR 0.88 (0.77-1.01) | OR 0.76 (0.49-1.13) | HR 0.84 (0.61-1.15) |
| **Coronary ICU (637 patients)** | OR 1.14 (0.95-1.36) | OR 1.12 (0.69-1.76) | HR 1.06 (0.65-1.72) |

## Table S7: Results using AIPW, TMLE and overlap weighting in the population with ROX ≤4.88 at eligibility. AIPW: Augmented inverse probability weighting, TMLE: Targeted maximum likelihood.

|  | **30-day mortality** | **One-year mortality** |
| --- | --- | --- |
| AIPW | OR 0.82 (95% CI 0.68-0.99) | OR 0.81 (95% CI 0.68-0.97) |
| TMLE | OR 0.87 (95% CI 0.59-1.22) | OR 0.79 (95% CI 0.52-1.13) |
| Overlap weighting | HR 0.94 (0.76-1.17, p =0.58) | HR 0.93 (95% CI 0.78-1.12, p=0.47) |

## Table S8: Results at one-year using overlap weights for the first hour of observation and for the remaining hours (hours 2 to 48). This was done since overlap was significantly worse from hour 2 and onwards.

|  | **30-day mortality** | **One-year mortality** |
| --- | --- | --- |
| **1^st^ hour** | HR 0.94 (95% CI 0.76-1.17, p =0.59) | HR 0.90 (95% CI 0.76-1.07, p =0.24) |
| **2^nd^ to 48^th^ hour** | HR 0.91 (95% CI 0.73-1.14, p =0.41) | HR 0.97 (95% CI 0.82-1.16, p =0.75) |

## Table S9: Complete-case analyses: 1) without any type of imputation including last observation carried forward and 2) allowing last observation carried forward for SOFA score items.

|  | **One-year mortality** |
| --- | --- |
| **Complete-case 1** | HR 0.70 (95% CI 0.55-0.89, p =0.003) |
| **Complete-case 2** | HR 0.82 (95% CI 0.69-0.97, p =0.02) |

## Table S10: ICU and hospital length of stay in the whole cohort and in survivors only. Data is presented as intubated in first place.

|  | **ICU length of stay** | **Hospital length of stay** |
| --- | --- | --- |
| **Whole cohort** | 5 (IQR 3-10) vs 4 (2-6) days (median difference 2 days, 95% CI 1-4 days). | 12 (IQR 6-19) and 10 (IQR 6-16) days (median difference 2 days, 95% CI 0-3 days). |
| **Survivors** | 8 days (IQR 4-14) vs 4 (IQR 3-5) days (median difference 5 days, 95% CI 1-9 days). | 12 (IQR 8-18) and 9 (IQR 6-15) days (median difference 3 days, 95% CI 2-5 days). |


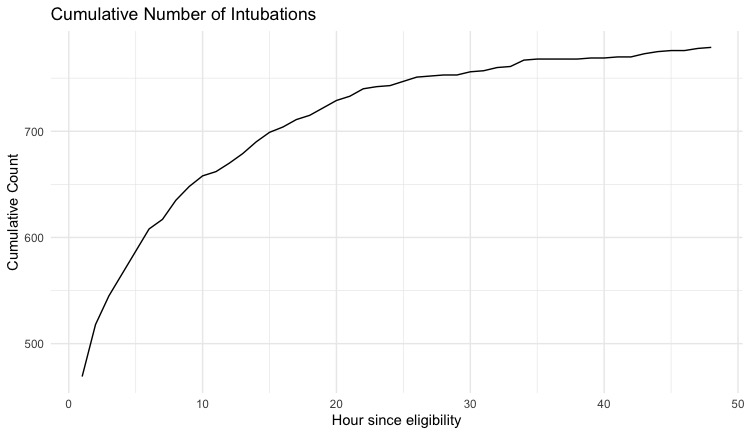


Figure S1. Cumulative number of intubations over the study period. The moment of eligibility was defined as hour 1 and patients were followed for 48 hours afterwards.


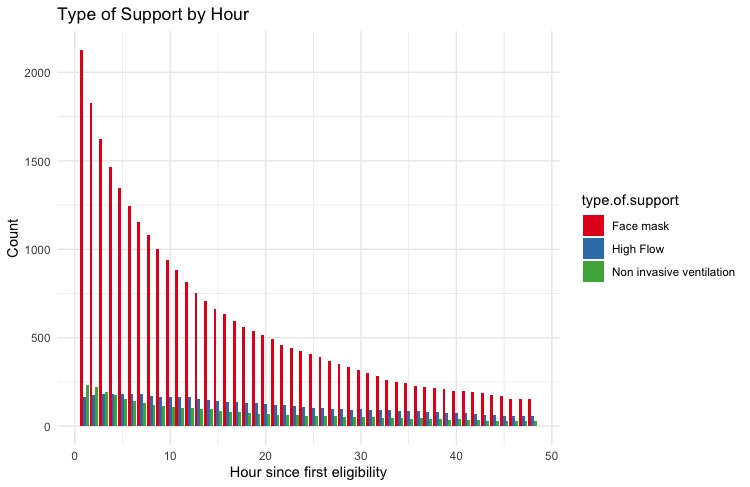


## Figure S2. Distribution of non-invasive support received at each hour after eligibility had been met.


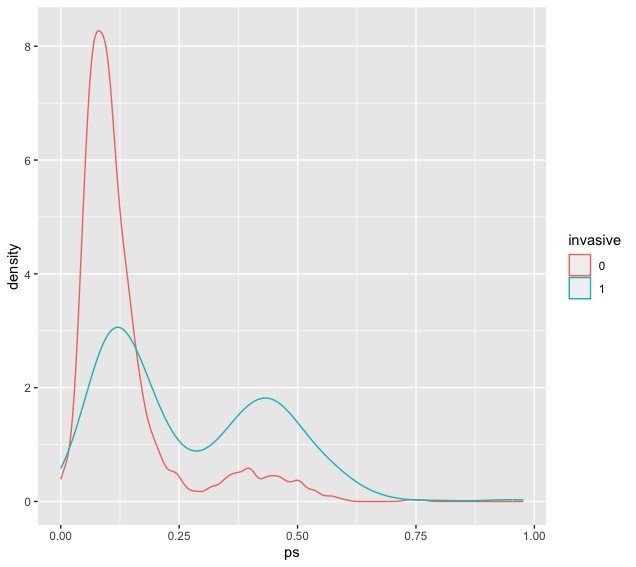


Figure S3: Propensity score distribution by groups of treatment at hour 1. Intubated observations represent 469 cases (15% of the total number) of observations at hour 1 and 60% of intubations during the whole study period.


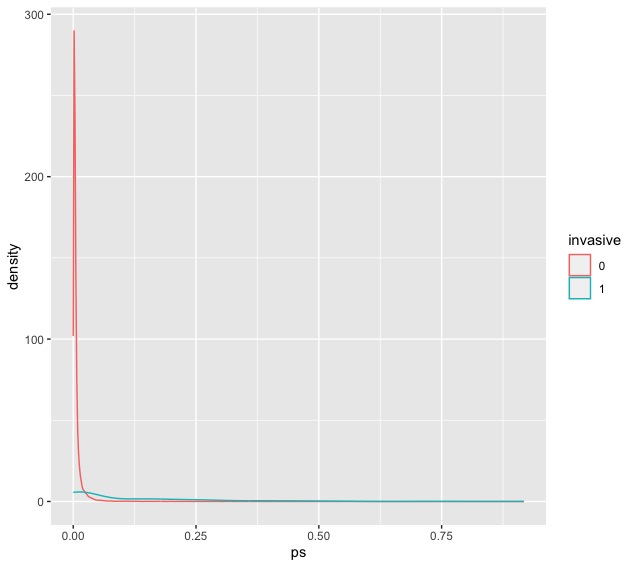


Figure S4: Propensity score distribution by groups of treatment from hour 2 and onwards. Intubated observations represent 310 cases (less than 0.1%) of the total number of observations.

# **REFERENCES**

1. Johnson AEW, Bulgarelli L, Shen L, et al. MIMIC-IV, a freely accessible electronic health record dataset. *Sci Data 2023 101*. 2023;10(1):1-9. doi:10.1038/s41597-022-01899-x

2. Cole SR, Hernán MA. Constructing inverse probability weights for marginal structural models. *Am J Epidemiol*. 2008;168(6):656-664. doi:10.1093/AJE/KWN164

3. Coudroy R, Frat JP, Girault C, Thille AW. Reliability of methods to estimate the fraction of inspired oxygen in patients with acute respiratory failure breathing through non-rebreather reservoir bag oxygen mask. *Thorax*. 2020;75(9):805-807. doi:10.1136/THORAXJNL-2020-214863

4. Austin PC. A tutorial and case study in propensity score analysis: An application to estimating the effect of in-hospital smoking cessation counseling on mortality. *Multivariate Behav Res*. 2011;46(1):119-151. doi:10.1080/00273171.2011.540480

5. Desai RJ, Franklin JM. Alternative approaches for confounding adjustment in observational studies using weighting based on the propensity score: A primer for practitioners. *BMJ*. 2019;367. doi:10.1136/bmj.l5657

6. Stensrud MJ, Hernán MA. Why Test for Proportional Hazards? *JAMA - J Am Med Assoc*. 2020;323(14):1401-1402. doi:10.1001/jama.2020.1267

7. Haneuse S, Vanderweele TJ, Arterburn D. Using the E-Value to Assess the Potential Effect of Unmeasured Confounding in Observational Studies. *JAMA - J Am Med Assoc*. 2019;321(6):602-603. doi:10.1001/jama.2018.21554
